# Cost-effectiveness of dihydroartemisinin-piperaquine compared with artemether-lumefantrine for treating uncomplicated malaria in children at a district hospital in Tanzania

**DOI:** 10.1186/1475-2875-13-363

**Published:** 2014-09-15

**Authors:** Amani T Mori, Frida Ngalesoni, Ole F Norheim, Bjarne Robberstad

**Affiliations:** Centre for International Health, Department of Global Public Health and Primary Care, University of Bergen, P.O. Box 7804, 5020 Bergen, Norway; Muhimbili University of Health and Allied Sciences, P.O. Box 65001, Dar es Salaam, Tanzania; Ministry of Health and Social Welfare, P.O. Box 9083, Dar es Salaam, Tanzania

**Keywords:** Tanzania, Dihydroartemisinin-piperaquine, Artemether-lumefantrine, Malaria, Cost-effectiveness, Markov model, Disability adjusted life years

## Abstract

**Background:**

Dihydroartemisinin-piperaquine (DhP) is highly recommended for the treatment of uncomplicated malaria. This study aims to compare the costs, health benefits and cost-effectiveness of DhP and artemether-lumefantrine (AL) alongside “do-nothing” as a baseline comparator in order to consider the appropriateness of DhP as a first-line anti-malarial drug for children in Tanzania.

**Methods:**

A cost-effectiveness analysis was performed using a Markov decision model, from a provider’s perspective. The study used cost data from Tanzania and secondary effectiveness data from a review of articles from sub-Saharan Africa. Probabilistic sensitivity analysis was used to incorporate uncertainties in the model parameters. In addition, sensitivity analyses were used to test plausible variations of key parameters and the key assumptions were tested in scenario analyses.

**Results:**

The model predicts that DhP is more cost-effective than AL, with an incremental cost-effectiveness ratio (ICER) of US$ 12.40 per DALY averted. This result relies on the assumption that compliance to treatment with DhP is higher than that with AL due to its relatively simple once-a-day dosage regimen. When compliance was assumed to be identical for the two drugs, AL was more cost-effective than DhP with an ICER of US$ 12.54 per DALY averted. DhP is, however, slightly more likely to be cost-effective compared to a willingness-to-pay threshold of US$ 150 per DALY averted.

**Conclusion:**

Dihydroartemisinin-piperaquine is a very cost-effective anti-malarial drug. The findings support its use as an alternative first-line drug for treatment of uncomplicated malaria in children in Tanzania and other sub-Saharan African countries with similar healthcare infrastructures and epidemiology of malaria.

**Electronic supplementary material:**

The online version of this article (doi:10.1186/1475-2875-13-363) contains supplementary material, which is available to authorized users.

## Background

Malaria is an infectious disease which disproportionately affects pregnant women and children under the age of five years, and the disease is a major health problem in sub-Saharan Africa. In 2012, an estimated 627,000 deaths occurred due to malaria globally, mostly in African children under the age of five years [1]. Malaria accounts for 3.3% (82,685,000) of all Disability Adjusted Life Years (DALYs) and is ranked seventh among the top leading causes of DALYs globally [2]. Over the years, countries in sub-Saharan Africa have repeatedly changed their treatment policies in response to parasite resistance to monotherapy anti-malarials [3]. Recently, more expensive artemisinin-based combination therapy (ACT) has been recommended and have become increasingly common as first-line regimens against *Plasmodium falciparum* malaria [1, 3].

The World Health Organization (WHO) recommends several artemisinin-based combinations for the treatment of uncomplicated malaria, including artesunate-sulphadoxine-pyrimethamine (ASSP), artesunate-amodiaquine (ASAQ), artesunate-mefloquine (ASMQ), artemether-lumefantrine (AL) and dihydroartemisinin-piperaquine (DhP) [4]. The newest ACT on this list is DhP, which has been proved to be more effective [5, 6], but is unfortunately also more expensive than AL, which is currently the most commonly used ACT in sub-Saharan Africa. Despite being more expensive, DhP has been recommended as a first-line or second-line alternative treatment for uncomplicated malaria [7–13].

In 2007, Tanzania changed its malaria treatment guidelines and adopted the use of AL as the first-line treatment for uncomplicated *P. falciparum* malaria to replace SP [14]. In 2013, the standard treatment guidelines were updated and DhP was officially adopted as the second-line drug for uncomplicated malaria [15]. AL has been shown to be a highly cost-effective first-line drug for the treatment of uncomplicated malaria [16, 17], but the cost-effectiveness evidence for DhP compared to AL is very limited [18].

Several countries in sub-Saharan Africa have officially adopted the use of DhP for the treatment of uncomplicated malaria [19, 20], and many others in the region are also contemplating this change. New drugs are typically more expensive than the existing alternatives: hence good trial results alone should not guarantee their inclusion in treatment guidelines as the additional health benefits may not be worth the extra costs. Pharmacoeconomic analyses are increasingly being used to generate evidence for decision-making in developing countries [21]. Therefore, this study aims to compare the costs, health benefits and cost-effectiveness of DhP and AL alongside “do-nothing” as a baseline comparator in order to consider the appropriateness of DhP as a first-line anti-malarial drug for children in Tanzania.

## Methods

### Decision model

**Figure 1:**
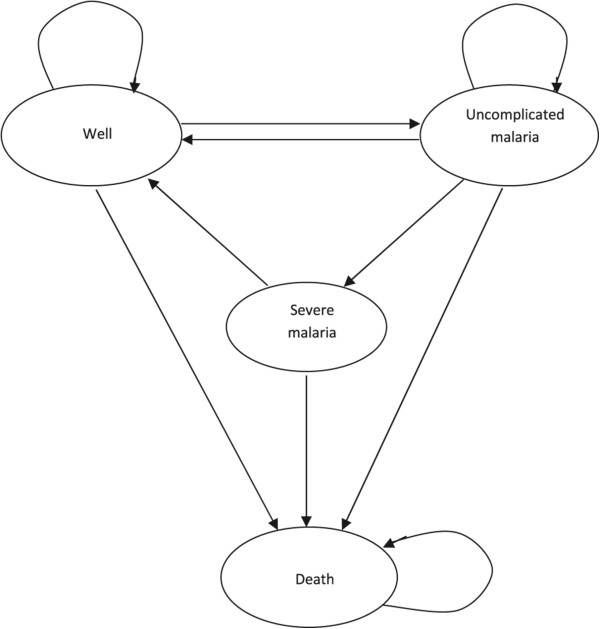
**State-transition diagram of the model.**

Cost-effectiveness was analysed using a Markov decision model with four mutually exclusive health states: “well”, “uncomplicated malaria”, “severe malaria” and “death” (Figure 1). Newborn children are assumed to be protected from malaria through breastfeeding, and enter the model when they are six months old in a “well” state. In the model, they are tracked until they are five years old, after which they are assumed to have gained sufficient clinical immunity against malaria [22, 23]. During this time, children move between the health states in one-week cycles depending on risk factors, access to and effectiveness of anti-malarial treatments.

The model assumes that children first develop uncomplicated malaria, from which they may recover and return to the “well” state, or they may progress to “severe malaria”, which requires hospitalization. “Death” is an absorbing health state, which may occur spontaneously (i.e. background mortality) or as an outcome of severe malaria. In each cycle the model captures and accumulates costs and utilities related to the patient’s health state. Probabilistic Sensitivity Analyses (PSA) were based on a Monte Carlo simulation with 10,000 iterations using TreeAge Pro^©^ 2014 software.

### Collection of cost data

Cost data from a provider’s perspective was collected at Mwananyamala Hospital in Dar- es Salaam region, from August to November 2012. This is an urban, district-level public hospital with about 400 beds and 400,000 visits per year. Costs were collected for the treatment of both uncomplicated and severe malaria in order to capture the additional costs for patients who develop severe malaria after unsuccessful treatment with the first-line drugs. A district hospital was chosen because it is the lowest level at which severe malaria can be managed effectively within the Tanzanian healthcare system. Costs represent the expenditures incurred during the financial year that ended on June 30^th^ 2012 and were collected using an ingredient approach [24]. Costs were collected in the local currency and converted to US dollars (US$ 1 = 1,578 Tanzanian shillings) [25].

Four service centres were identified; namely the general outpatient department, general paediatric ward, pharmacy and the laboratory. Support departments, which included general administration and transportation, were categorized as overheads. Costs for resources which last longer than a year were categorized as capital costs and included furniture, equipment and motor vehicles. Recurrent costs were those incurred on resources that are purchased regularly and used up in the course of a year, and include salaries rental charges, utilities and supplies [26].

Cost data were recorded in a pre-tested questionnaire which was designed to capture all the necessary data, including the types and quantities of items, their sources, prices and allocation base. Functioning capital items were identified, counted and valued using their assumed replacement market prices. The price catalogue from the Medical Stores Department (MSD) was used to value medical items and supplies [27]. Capital costs were annuitized at a discount rate of 12% as recommended by the Bank of Tanzania [25] and their useful life years were adopted from the WHO-CHOICE Project [28].

Staff members were identified and interviewed in order to discover their monthly earnings, including gross salary and other standard remunerations. Salary scales and remunerations were cross-checked and validated by the hospital secretary. Personnel costs attributable to malaria were calculated by multiplying total staff monthly earnings by the percentage of their time devoted to malaria. For the buildings, floor spaces were measured and valued as per the square metre rental charges recommended by the National Housing Corporation.

The Global Fund’s maximum manufacturer prices for ACTs that are financed through the Affordable Medicines Facility-malaria (AMFm) was used to estimate the mean cost of a course of treatment with AL and DhP [29]. For AL the “6×2” tablet pack specified for children weighing 15–24 kg was used [13] and the “3×1” tablet pack for children weighing 13–24 kg was used for DhP [30]. These prices were inflated by 10% to account for freight and insurance costs [31] and further by a domestic margin factor of 1.43 to represent local opportunity costs [32]. Prices of all the other drugs used in the management of malaria were taken from the MSD’s Price Catalogue.

Each service department was allocated a portion of the overhead costs proportional to its percentage contribution to the total allocation base by using the direct-allocation method [24]. For example, cleaning costs were allocated based on floor space. Allocation was difficult for some expenditure, such as electricity, medical supplies, stationery, which were paid for centrally but for which usage was not specified by the departments. Therefore, some of the overhead costs were equally distributed between the departments while others were allocated using an estimated weighted-allocation factor based on interviews with hospital management. For more details about personnel costs and rental charges, see Additional file 1.

The hospital has Health Management and Information System (HMIS) tools to keep records of all the attendances and diagnoses made during each year. However, because of poor recording, the attendances of malaria patients in the pharmacy or the laboratory could not be tracked. Therefore, the unit costs for the treatment of uncomplicated and severe malaria were calculated by dividing the total costs attributable to malaria for the service centres by the respective number of outpatients (7,076 cases) and hospitalized patients (1,263 cases) recorded in the HMIS tools during the year.

### Choice of health outcomes

Disability Adjusted Life Years (DALYs), which combines years of life lost due to premature death (YLL) and years of life lived with disabilities (YLD), was used as a measure of health outcomes [2]. Disability weights of 0.005 and 0.21 for mild and severe acute episodes of infectious diseases from the recent Global Burden of Disease study were applied for uncomplicated and severe malaria, respectively [33]. DALYs averted were calculated using standard methods [34] as a difference of DALYs lost with and without the intervention, based on a life expectancy of 57 years at age 5 for Tanzania [35]. Base case DALYs were discounted at 3%, without age-weighting. Results for age-weighted and undiscounted DALYs were reported in the scenario analysis.

### Interventions compared

The study compares DhP (the potential new standard of care) and AL (the existing standard of care) alongside “do nothing” as a baseline comparator. Both drugs are administered for three consecutive days, but AL should be given twice a day with high-fat meals [36] while DhP is given once a day without the requirement for fatty meals [37]. Because of its relatively simple dosage regimen, it is likely that compliance with and hence the effectiveness of DhP will be higher than that of AL in clinical settings. DhP also offers a longer patient protection from re-infection with malaria because piperaquine has a significantly longer elimination half-life of 3–4 weeks compared to the 4–6 days of lumefantrine [38]. The impact of high compliance with DhP is included in the base-case scenario of our model, while that of longer protection is not.

### Measurement of effectiveness

Patient compliance to treatment in routine clinical practice plays a key role in the effectiveness of anti-malarial therapies. Thus the effectiveness of each drug, E_ff_, was calculated by combining efficacy and compliance rates using the equation below:


Where E_o_ is the efficacy, C is the compliance rate and E_nc_ is the proportion of non-compliers for whom treatment is effective, assumed to be 10–30%, which has been employed in several other cost-effectiveness studies for ACT [39–41]. Efficacy data were extracted from a large, head-to-head, randomized clinical trial which was conducted among 6–59-month-old children in seven African countries with different malaria endemicities: Uganda, Zambia, Mozambique, Rwanda, Nigeria, Gabon and Burkina Faso. The study used the 28-day PCR-corrected cure rate of 97.3% for DhP and 95.5% for AL, from the intention-to-treat analysis [13].

Evidence on compliance to ACT is very limited and diverse [42, 43]; however, it has been reported that compliance to AL by “verified timely completion” ranges from 38 to 65% [43]. DhP is a new drug and evidence on its compliance is currently lacking. Since the potential benefit of its once-a-day dosage regimen consisting of only a few tablets is an improved compliance, a range of 60 to 80% was assumed in the base case analysis. This is a conservative assumption, considering that a compliance of 67–87% and 87.2–92.5% have been reported for co-blistered and fixed-dose ASAQ, among children in Tanzania and Madagascar, respectively [44, 45]. ASAQ has a once-a-day dosing schedule similar to that of DhP. An assumed compliance similar to that of AL was explored in a scenario analysis.

### Transition probabilities

Children enter the model in a “well” state, and can develop febrile episodes based on the estimated age-specific incidence rates shown in Table 1. All febrile children were assumed to be taken to the hospital for diagnosis, and 10.5% of the episodes were attributed to malaria [46]. Between 40 and 60% of children with uncomplicated malaria were assumed to have access to first-line drugs and the probability of cure depends on efficacy and compliance with treatment. Efficacies of AL and DhP were 95.5% and 97.3%, [13] and the base line compliance rates ranged between 38–65% for AL [43] and 60–80% for DhP. The remaining children were assumed to be treated with over-the-counter non-ACT anti-malarials, with effectiveness ranging from 40 to 60% [47, 48].

**Table 1:** **Parameters used in the economic model and their distributions**

Parameters	Estimates	Distributions	Sources
*Age-specific probabilities of death*			
Probability of dying between 0 and 1 year	0.0684 *±* 20%	Beta	[35]
Probability of dying between 1 and 5 years	0.0424 *±* 20%	Beta	[35]
Malaria-attributed deaths in under fives	11%	Point estimate	[49]
*Weekly incidences of fever episodes per child*			
Less than 12 months	0.106 *±* 20%	Beta	[50]
Age 12–23 months	0.144 *±* 20%	Beta	[50]
Age 24–35 months	0.105 *±* 20%	Beta	[50]
Age 36–47 months	0.087 *±* 20%	Beta	[50]
Age 48–59 months	0.06 *±* 20%	Beta	[50]
*Case fatality rates and other probabilities*			
Untreated severe malaria	60 (45–80%)	Beta	[51]
Treated severe malaria	10.9%	Beta	[52]
Early treatment failure leads to severe malaria	5 (3–7%)	Beta	[41]
Untreated malaria becomes severe	5 (3–7%)	Beta	[40]
Spontaneous recovery from uncomplicated malaria	15 (10–20%)	Beta	Assumed
% of febrile episodes attributed to malaria	10.5 *±* 20%	Beta	[46]
% of severe cases with access to inpatient care	80 *±* 20%	Beta	[53]
% of uncomplicated cases with access to AL	50 (40–60%)	Beta	Primary data
*Costs of treating malaria, by severity (US$/case)*			
Uncomplicated malaria	6.81 *±* 20%	Gamma	Primary data
Severe malaria	76.46 *±* 20%	Gamma	Primary data
*Drug costs (US$ per dose)*			
DhP: 40 mg Dh, 320 mg P (“3×1” pack)	1.46 *±* 20%	Gamma	[29]
AL: 20 mg A, 120 mg L (“6×2” pack)	1.31 *±* 20%	Gamma	[29]
Quinine Injection, 300 mg/ml (2 ml ampoule)	2.15 *±* 20%	Gamma	[27]
Diazepam Injection, 5 mg/ml (2 ml ampoule)	0.23 *±* 20%	Gamma	[27]
Diclofenac Injection 25 mg/ml (3 ml ampoule)	0.20 *±* 20%	Gamma	[27]
Dextrose 5% (500 ml bottle)	4.75 *±* 20%	Gamma	[27]
Ferrous Sulphate + Folic acid, 200 + 0.25 mg	0.30 *±* 20%	Gamma	[27]
Paracetamol Syrup 120 mg/5 ml	0.26 *±* 20%	Gamma	[27]
*Efficacy and compliance rates (%)*			
Efficacy of DhP	97.3 *±* 2.5%	Beta	[13]
Efficacy of AL	95.5 *±* 2.5%	Beta	[13]
Effectiveness of non-ACT anti-malarials	50 (40-60%)	Beta	[47, 48]
Compliance with AL	51 (38–65%)	Uniform	[43]
Compliance with DhP^a^	70 (60–80%)	Uniform	Assumed
Compliance with DhP^b^	51 (38–65%)	Uniform	Assumed
Non-compliers with ACTs who are cured	20 (10–30%)	Beta	[39–41]
*Other parameters*			
Disability weight for uncomplicated malaria	0.005 (0.033–0.081)	Beta	[33]
Disability weight for severe malaria	0.21 (0.139–0.298)	Beta	[33]
Discount rate	3%	Point estimate	[54]
Decision threshold (US$ per DALY averted)	150	Point estimate	[55]
Life expectancy at age 5 years	57	Point estimate	[35]
Sensitivity of Microscopy	71.3 (68.8–73.9%)	Beta	[56]
Specificity of Microscopy	92.8 (91.3–94.3%)	Beta	[56]

^a^Used in the base case analysis, ^b^Used in the scenario analysis.

In the “do-nothing” arm, between 3–7% of uncomplicated malaria cases progress to severe malaria [40], which has been estimated to have a case fatality rate ranging from 45 to 80% [51]. Between 10 to 20% of the uncomplicated malaria cases were assumed to recover spontaneously without treatment. In the DhP and AL arms, about 3–7% of the uncomplicated malaria cases progress to severe malaria in the event of treatment failure [41], of whom between 72–88% were assumed to have prompt access to inpatient care [53], which reduces case-fatality rate to 10.9% [52]. Besides malaria, children can also die of other causes at any state in the model based on adjusted age-specific probabilities of death taken from the Tanzanian Life Table [35].

### Sensitivity and specificity of the test

Bayesian method was used to incorporate the sensitivity and specificity parameters of the microscopic test in the model, which have been estimated to be 71.3% (95% CI: 68.8–73.9) and 92.8% (95% CI: 91.3–94.3), respectively [56]. Rate of adherence by clinicians to negative test results was estimated to range from 40 to 60% [56].

### Model simplifications

The model is a simplification of a complex disease with complex treatment-seeking behaviour and management practices. It is based on the following simplifying assumptions:

-  A child cannot move directly from a “well” to “severe malaria” state, but severe malaria is always a progression from uncomplicated malaria.
-  Uncomplicated malaria is not fatal, hence a child cannot move from “uncomplicated malaria” to the “death” state, except for deaths caused by other reasons (i.e. background mortality).
-  In the event of treatment failure, patients with uncomplicated malaria will repeatedly use the same first-line drug, which we assumed will still be effective.

### Uncertainty and sensitivity analyses

Uncertainties in parameters were included in the model by using probability distributions (Table 1). Maximum and minimum values for each parameter were taken from the literature and when these were not available, the mean values were varied by +/- 20% and efficacy data by +/- 2.5%. The gamma distribution was used to constrain costs on the [0,+∞] interval and the beta distribution to fix the probabilities on the [0,1] interval. Gamma and beta distributions were calculated using the method of moments [57]. Uncertainty in the PSA results is presented using a cost-effectiveness acceptability curve (CEAC). Sensitivity and scenario analyses were also performed to assess the influence of variations in the key parameters.

### Cost-effectiveness threshold

An intervention that produces more health benefits at a lower cost than the comparator is considered to be “strongly dominant” and cost-effective. If it is more costly but also more effective, it is considered cost-effective only when its incremental cost-effectiveness ratio (ICER) is less than the willingness-to-pay threshold. “Extended dominance” occurs when the ICER of an intervention is higher than that of the next most effective option [58]. A willingness-to-pay threshold of US$ 150 per DALY averted, which has been recommended as a cut-off point for low- and middle-income countries was applied [55].

### Ethics statement

This study was approved by the Ethical Review Committee of the Tanzania National Institute of Medical Research with clearance certificate no: NIMR/HQ/R.8a/Vol.IX/1362. The District Medical Officer in charge of Kinondoni and the management at Mwananyamala Hospital also gave permission to conduct the costing study. The interviewed health workers each provided written informed consent to participate in the study.

## Results

### Unit costs of treatment

Table 2 presents the estimated unit costs of treating cases of uncomplicated and severe malaria with the associated co-morbidities at an urban district-level hospital in Tanzania. For uncomplicated malaria, the cost per episode was US$ 8.40 with AL and US$ 8.54 with DhP. For severe malaria, the hospitalization cost per episode was estimated to be US$ 83.86.

**Table 2:** **Unit costs (US$) for outpatient and inpatient care**

Out-patient care for uncomplicated malaria							
		Service centres			Cost	Unit costs	
Cost category	Items	Outpatient	Pharmacy	Laboratory	Total	AL	DhP
Recurrent	Antimalaria drugs	-	-	-	-	1.31	1.46
	Other drugs					0.26	0.26
	Personnel	22,988	7,748	6,066	36,802	5.20	5.20
	Rental of buildings	1,131	538	1,647	3,316	0.47	0.47
	Utilities	1,533	1,368	1,539	4,440	0.63	0.63
	Supplies	700	699	1,558	2,957	0.42	0.42
Capital	Equipment	31	89	108	228	0.03	0.03
	Motor vehicles	70	25	25	121	0.02	0.02
	Furniture	162	109	69	340	0.05	0.05
**Total unit costs**						8.40	8.54
**Inpatient care for severe malaria**							
**Cost category**	**Items**	**Paediatric ward**	**Pharmacy**	**Laboratory**	**Total**	**Unit costs**	
Recurrent	Drugs	-	-	-	-	7.40	
	Personnel	75,895	1,202	845	77,942	61.71	
	Rental of buildings	8,012	717	305	9,034	7.15	
	Utilities	3,780	494	285	4,559	3.61	
	Supplies	2,265	162	288	2,715	2.15	
Capital	Equipment	442	21	20	482	0.38	
	Motor vehicles	457	3	5	465	0.37	
	Furniture	1,319	39	13	1,371	1.09	
**Total unit costs**						83.86	

### Cost-effectiveness analysis

Table 3 presents the base-case analysis, for which the model predicts that DhP is more cost-effective than AL, with an ICER of US$ 12.40 per DALY averted. AL was eliminated in the base-case analysis because it was extendedly dominated by DhP, therefore, the base-case ICER value represents the comparison of DhP to a do nothing strategy. In the scenario assuming a lower compliance, similar to that of AL, ranging from 38–65%, AL was more cost-effective than DhP with an ICER of US$ 12.54 per DALY averted versus US$ 101.52 per DALY averted.

**Table 3:** **Base-case cost-effectiveness analysis**

Strategy	Cost (US$)	DALYs	Incremental cost	Incremental DALYs averted	ICER
No treatment	0.00	17.60	0.00	0.00	0.00
AL	165.42	4.47	165.42	13.13	Extendedly dominated
DhP	166.22	4.22	0.80	0.25	12.40

### Incremental cost-effectiveness scatter plot

**Figure 2:**
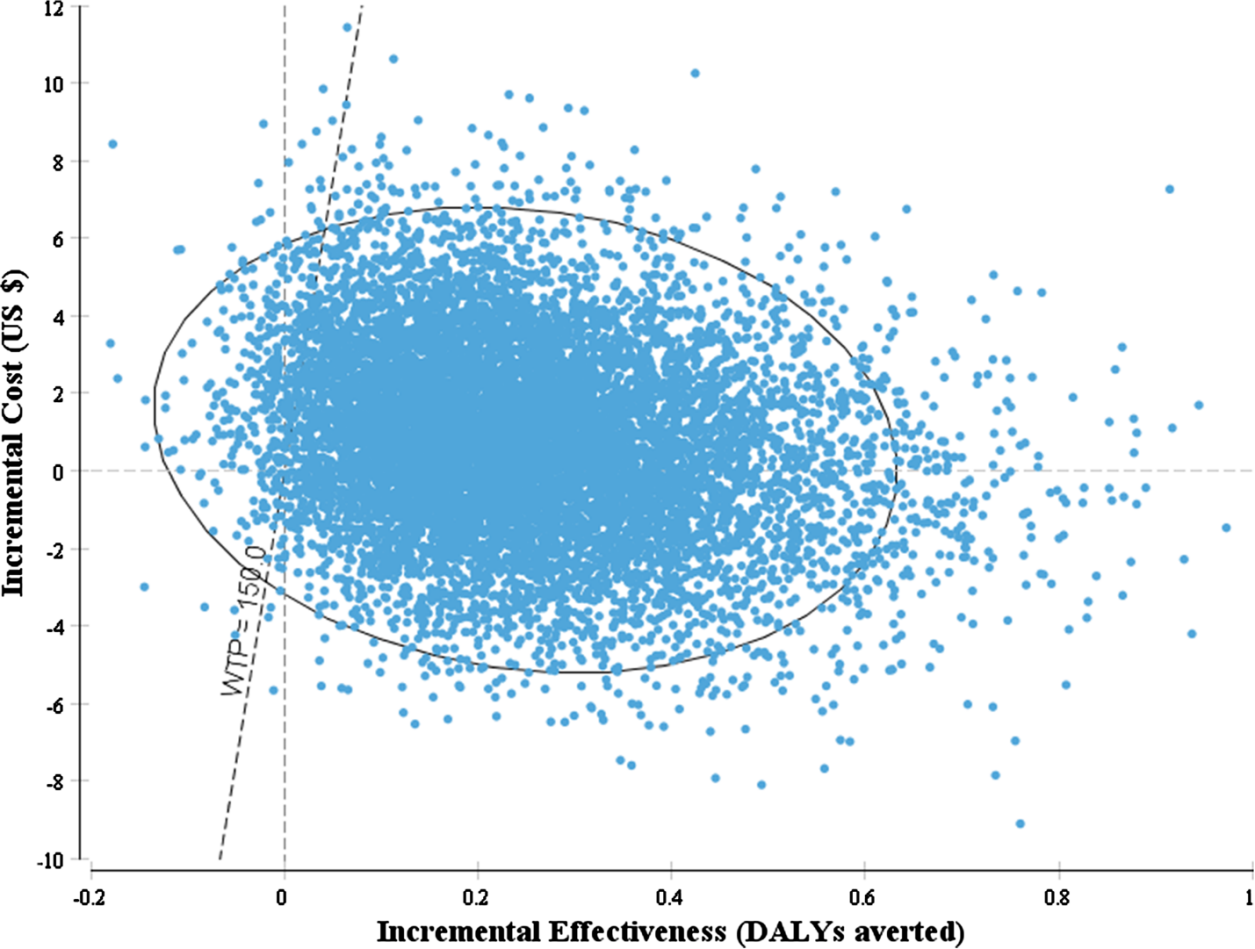
**Incremental cost-effectiveness scatter plot DhP versus AL.** Key: The dots represent incremental cost-effect pairs for DhP versus AL for 10,000 Monte Carlo simulations. The dotted line represents a willingness-to-pay threshold of US$ 150 per DALY averted.

Figure 2 shows the base-case ICE scatter plot of DhP versus AL. The model predicts that DhP is cost-effective in 97% of the simulations and dominated by AL in 2% of the simulations, at a willingness-to-pay threshold of US$ 150 per DALY averted. With a compliance of 38–65%, DhP was cost-effective in 51% of the simulations and dominated by AL in 37% of the simulations.

### Cost-effectiveness acceptability curve

**Figure 3:**
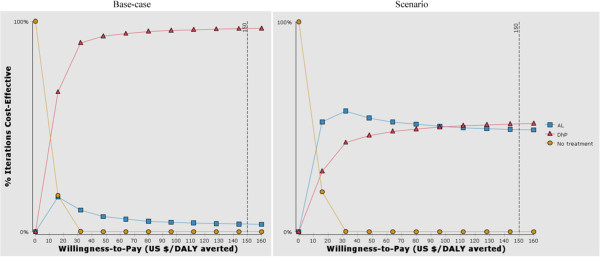
**Cost-effectiveness acceptability curves.**

Figure 3 shows the cost-effectiveness acceptability curves (CEAC) for the base-case and scenario analyses of DhP compared to AL. For the base-case, the probability of DhP being cost-effective was 97% at the willingness-to-pay threshold of US$ 150 per DALY averted. In the scenario analysis where we assumed the compliance with DhP to be 38–65%, the probability of DhP being cost-effective was 51% compared to 49% for AL at the same willingness-to-pay threshold of US$ 150 per DALY averted

### Characterizing uncertainty

**Figure 4:**
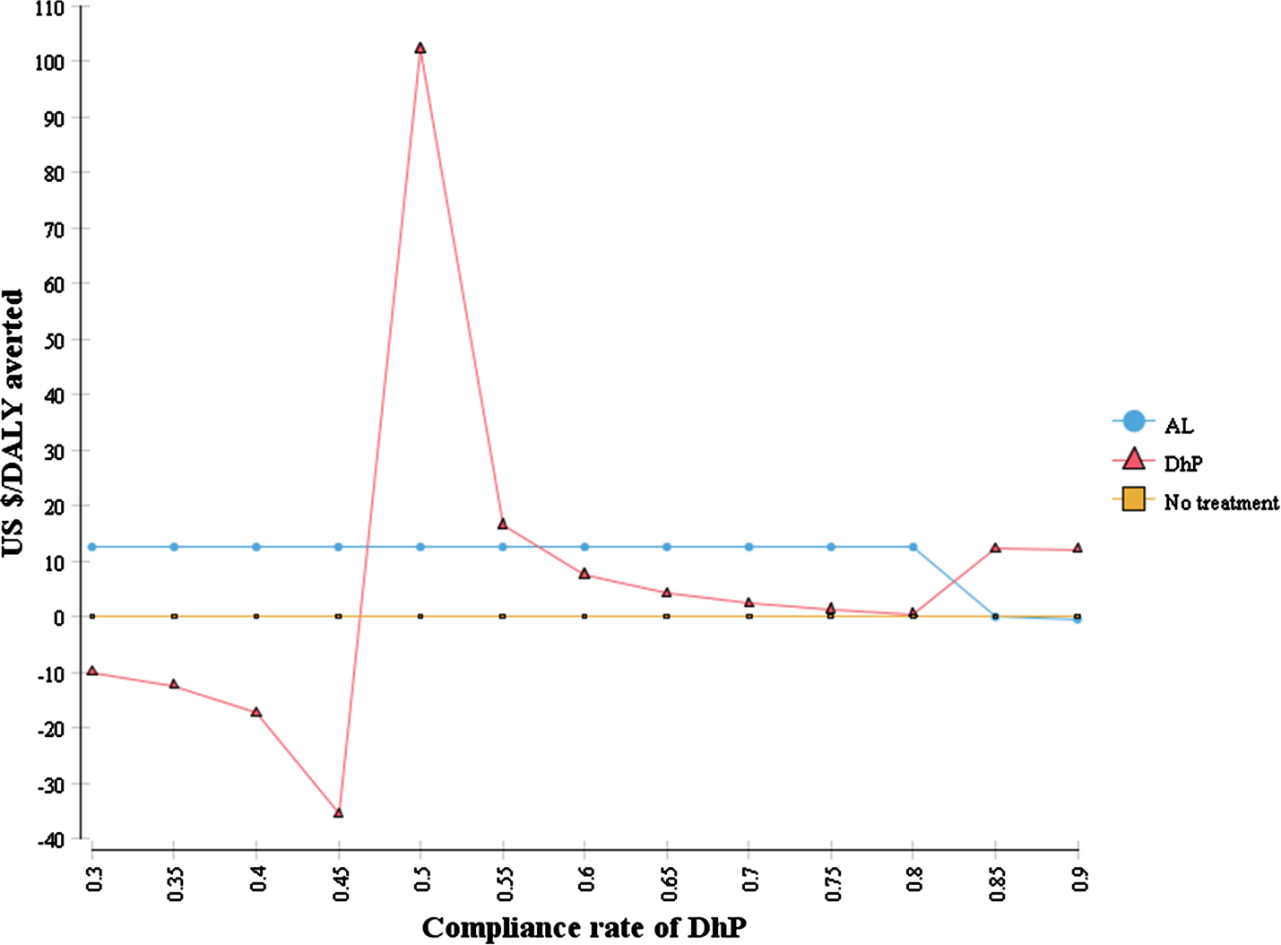
**Sensitivity of ICER to variations in the compliance rates for DhP.**

**Figure 5:**
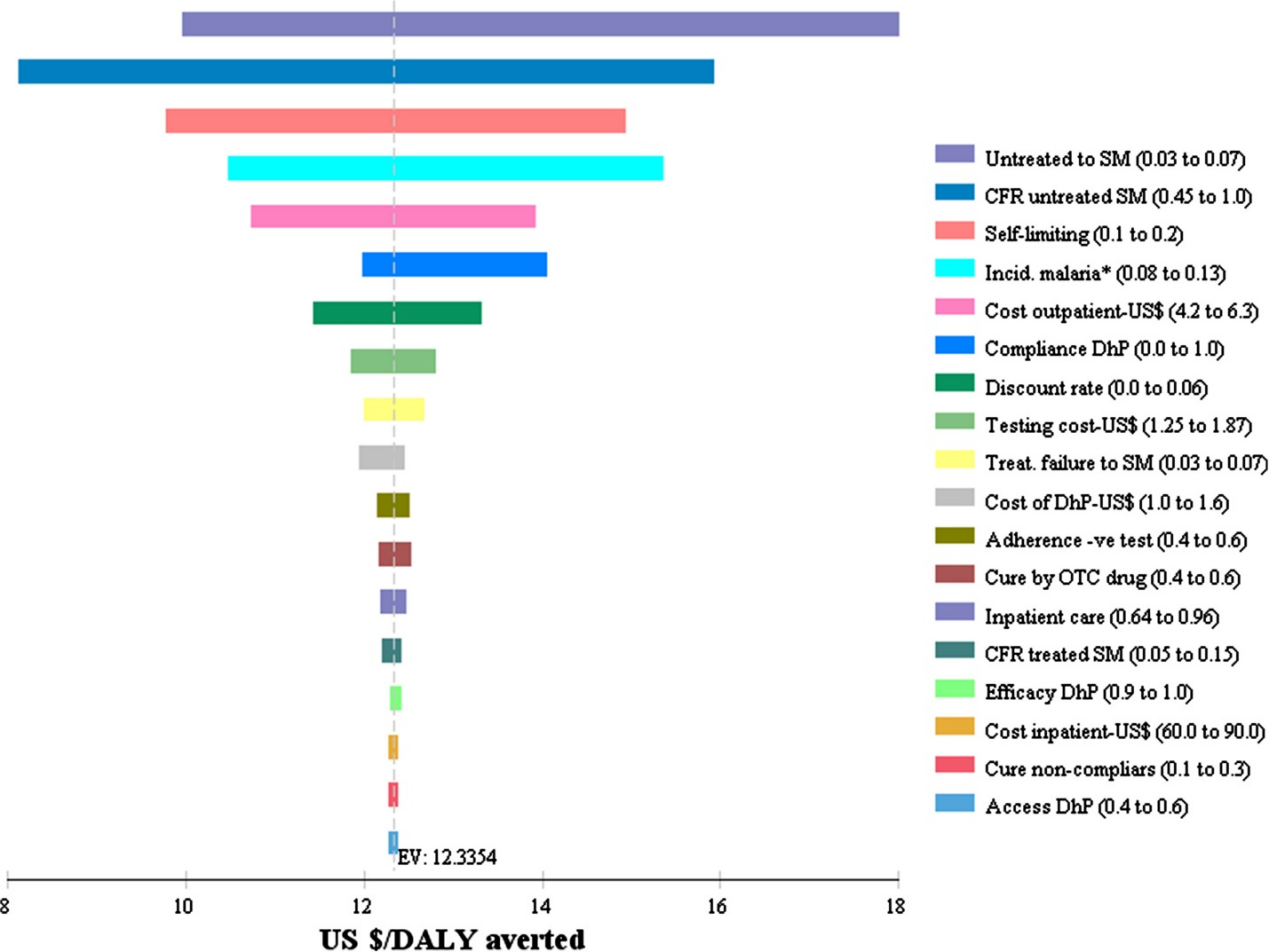
**ICER tornado diagram of DhP compared to “do-nothing”.** Key: *Incidence rates as percentage of febrile episodes. Unless otherwise indicated, the numbers in the brackets represent probabilities

One-way sensitivity analyses were conducted to assess the influence of plausible variations of key parameters on cost-effectiveness of DhP versus AL. The result shows that the cost-effectiveness of DhP relies on the assumption that it has a higher compliance rate than AL, for which the evidence is weak. This is illustrated in Figure 4, which shows that when the compliance with DhP is assumed to be less than 50% it produces fewer health benefits at higher costs than AL (strongly dominated) and at between 50 and 56% it is less cost-effective than AL. When compliance exceeds a threshold of 57%, DhP becomes the cost-effective strategy by extended dominance. Above 85%, DhP produces more health benefits at a lower cost than AL (strong dominance). Note that the compliance rate for AL was held constant at 51%.Figure 5 shows a tornado diagram which ranks the parameters in the order of their decreasing influence on the base-line ICER value. In the diagram, DhP was compared to “do-nothing” because AL was eliminated in the analysis due to extended dominance. Uncertainties in parameters describing the natural history of malaria were the most influential on the ICER value. This includes the probability of progression to severe malaria (Untreated to SM), case fatality rate for severe malaria (CFR untreated SM) and the probability of “self-limiting” uncomplicated malaria. The cost-effectiveness of DhP increases with an increase in the values of the first two parameters but decreases with an increase in the probability of self-limiting malaria. The cost-effectiveness of DhP also increases with an increase in the incidence rates of malaria, making it a good choice in high-transmission areas.

### Two-way sensitivity analysis

**Figure 6:**
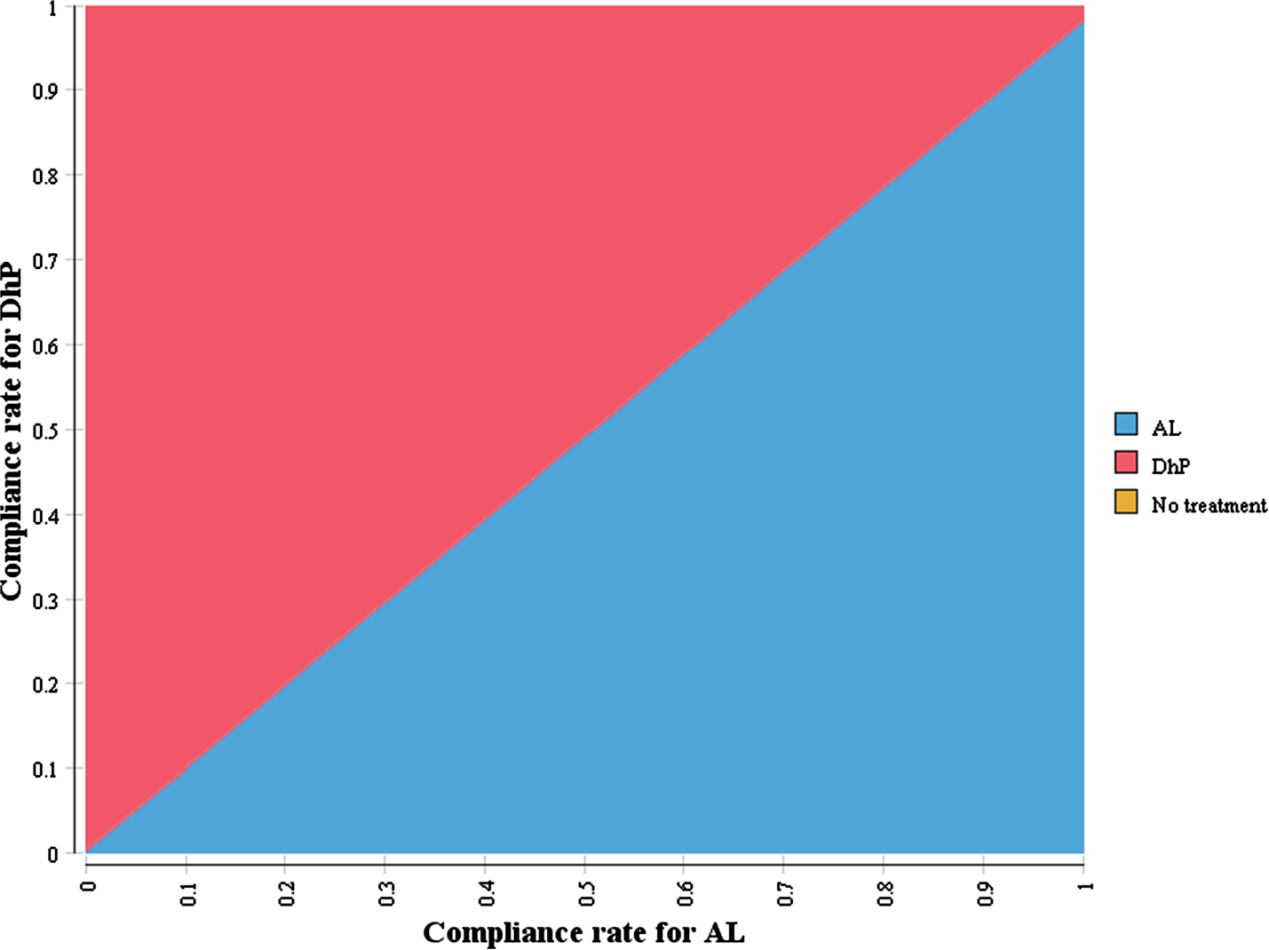
**Two-way sensitivity analysis of compliance rates for DhP and AL.**

The existing evidence for compliance with AL is very diverse. We therefore performed a two-way sensitivity analysis (Figure 6), to determine various combinations of compliance rates at which the two drugs were cost-effective, at a willingness-to-pay threshold of US$ 150 per DALY averted. This shows that even when compliance is perfect for both drugs, DhP remains slightly more cost-effective than AL.

### Impact of age-weighting and discounting

In the base-case analysis, DALYs were calculated without age-weighting and with a discount rate of 3%. When DALYs were not discounted, the ICER value of DhP compared to “do-nothing” in the deterministic analysis decreased from US$ 12.33 to 10.80 per DALY averted. Age-weighting assigns different values to time lived at different ages and when it was applied the ICER increased from US$ 12.33 to 18.00 per DALY averted. None of these choices of method had any influence on the conclusions.

## Discussion

This study has shown that DhP is a cost-effective anti-malarial drug with an incremental cost-effectiveness ratio of US$ 12.40 per DALY averted compared to AL. This finding is higher than the US$ 6.23 per DALY averted that was predicted by the Committee on the Economics of Anti-malarial Drugs, which compared ACT with “do-nothing”, from the provider’s perspective [40]. The ICER is well below all common rules of thumb for cost-effectiveness, including the GDP per capita for each DALY averted recommended by the WHO [59] and the US$ 150 per DALY averted suggested for low- and middle-income countries [55]. Therefore, adequate and timely provision of DhP can be considered a highly cost-effective treatment for uncomplicated malaria.

DhP is currently more expensive than AL and hence any decision to adopt it nationwide as a first-line drug will have significant budget implications. However, DhP has two major advantages over AL that make it an attractive weapon in the fight against malaria. Firstly, it has a relatively simple once-a-day, three-day dosage regimen and a bioavailability that does not require fat-rich meals [37]. This is likely to increase adherence to treatment, which will minimize wastage and improve therapeutic outcomes. Secondly, DhP has a long elimination half-life, which may give it a prolonged post-treatment prophylactic effect that would help to reduce future costs from recurrent infections [18].

In the base-case analysis, DhP was a dominant strategy based on the assumption that it has a compliance rate higher than that of AL; unfortunately, this has not been documented in clinical practice. Since the two drugs have similar safety profiles [13], and taking into account the complex dosage regimen and the pill burden of AL, it is unlikely that the compliance rate for DhP will be lower than that of AL. In addition, the prolonged post-treatment prophylactic effect of DhP, which we did not consider in the analysis, would increase its cost-effectiveness. A recent study has shown that DhP was strongly dominant over AL with a probability of 90%, by modelling the differences in post-treatment prophylactic effect of the two drugs [18].

DhP has received regulatory approval from the European Medicines Agency (EMA) and can now be procured with donor funds [60], at an affordable maximum price of less than US$ 1 per dose [29]. Sigma-Tau, the manufacturer of DhP (Eurartesim^®^), in collaboration with Medicine for Malaria Venture, are also developing a new water-dispersible formulation for children under the age of five years [61]. With generic competition, the price of DhP is likely to decrease even further over the coming years.

Even though DhP is a very promising long-acting anti-malarial drug, concerns have been raised about its residual drug levels as a potential risk for the emergence of resistance, especially in high transmission areas [62, 63]. A reliable surveillance system is therefore needed to monitor its therapeutic efficacy [13]. Several studies have also shown that the administered dosage and the resulting plasma concentrations are the most important predictors of treatment failures in children treated with DhP [64, 65]. Thus, malaria experts have suggested increasing the minimum dosage of piperaquine recommended by the WHO from 48 to 59 mg/kg in order to achieve desirable plasma concentrations [64].

Presumptive treatments and non-adherence to negative test results is another common challenge facing the deployment of expensive drugs like DhP in endemic countries. Studies in Tanzania have shown that malaria is highly over diagnosed and non-adherence to negative test results may be as high as 53% [56]. The WHO’s malaria report of 2011 showed that perfect compliance with negative test results would save US$ 68 million by eliminating the unnecessary use of ACT in the public sector in Africa [66].

### Limitations

The study used the two-week self-reported prevalence of fever from a national survey to estimate the weekly incidence rates of febrile episodes in children [50]. This approach can overestimate or underestimate the actual incidence rates given the seasonal variation of fever episodes and when counting is not precise due to the overlapping of fevers during the two-week period. This is, however, a preferred approach in the absence of systematically collected data about the annual incidence rates of febrile episodes [66].

Pragmatic costing studies are difficult to undertake in low-income countries because resource use and attendances at specific departments are not always properly documented. Therefore, we did not include costs for consumables, such as cannulas, syringes, cotton wool and infusion sets. It was also very challenging to allocate overhead costs to service departments, and this forced us to use weighted factors. It was also difficult to adequately calculate unit costs for laboratory and pharmacy services because attendances at these units were not properly recorded. Therefore, the estimated unit costs may have underestimated the actual treatment costs for uncomplicated and severe malaria.

The study was conducted from a provider’s perspective without including a more comprehensive societal perspective. Unlike DhP, the use of AL is associated with greater costs due to the requirement for fat-rich meals to optimize its bioavailability. A societal perspective may, therefore, increase treatment costs relatively more for AL, thus favouring DhP in the cost-effectiveness analysis.

The study was focused on health losses due to malaria only, therefore, DALYs lost from the associated co-morbidities of severe malaria such as anemia, convulsions and long-term neurological injury were not included in the model. Their inclusion would have favoured DhP in the cost-effectiveness analysis, because it is relatively more effective than AL due to its high efficacy and compliance rates.

### Generalizability

The study used AL, which is the current first-line anti-malarial drug against uncomplicated malaria in many malaria-endemic countries, as a comparator. The drug prices also include freight and insurance charges as well as local administrative costs, which to a large extent accommodate uncertainties in supplier prices. Given that the results were robust to plausible variations in all the key parameters, they are likely to be relatively generalizable to other settings with similar healthcare infrastructures and malaria epidemiology.

## Conclusion

DhP is a very cost-effective anti-malarial drug. The findings support its use as an alternative first-line drug for treating uncomplicated malaria in children in Tanzania and other sub-Saharan African countries with similar healthcare infrastructures and malaria epidemiology. A number of countries in malaria-endemic areas are currently considering the adoption of DhP in their malaria treatment guidelines. Therefore, policy-makers in these countries should employ this evidence in order to make informed decisions about allocating their limited resources to competing healthcare interventions.

## Electronic supplementary material

Additional file 1: **Personnel costs and rental charges.** (DOCX 27 KB)

## Abbreviations

ACT: Artemisinin-based combination therapies
AL: Artemether-lumefantrine
AMFm: Affordability medicines facility-malaria
DALY: Disability adjusted life years
DhP: Dihydroartemisinin-piperaquine
MSD: Medical stores department.
