# Supplementary material for: Cost-effectiveness of dihydroartemisinin-piperaquine compared with artemether-lumefantrine for treating uncomplicated malaria in children at a district hospital in Tanzania
Source: Malar J. 2014 Sep 15;13:363. doi: 10.1186/1475-2875-13-363 (PMC4171550; doi:10.1186/1475-2875-13-363)
Supplement: Supplementary file 1 — Additional file 1: Personnel costs and rental charges. (DOCX 27 KB) [file 12936_2014_3392_MOESM1_ESM.docx]

A: Direct Personnel costs

| 1. Paediatric ward personnel cost (US $) | | |  |  |  |
| --- | --- | --- | --- | --- | --- |
| No. | Cadre and qualification | full time equivalent | Monthly Gross salary | Monthly Benefits/allowance | Annual Earnings |
| 1 | MO | 1 | 889.1 | 126.7 | 12,190.1 |
| 2 | MO | 1 | 889.1 | 126.7 | 12,190.1 |
| 3 | MO | 1 | 737.9 | 126.7 | 10,375.7 |
| 4 | MO | 1 | 747.8 | 114.1 | 10,342.2 |
| 5 | Specialist | 1 | 1,160.3 | 221.8 | 16,585.6 |
| 6 | AMO | 1 | 914.4 | 114.1 | 12,342.2 |
| 7 | AMO | 1 | 914.4 | 114.1 | 12,342.2 |
| 8 | Intern | 1 | 485.4 | 95.1 | 6,965.8 |
| 9 | Intern | 1 | 485.4 | 82.4 | 6,813.7 |
| 10 | RN | 1 | 604.4 | 63.4 | 8,013.7 |
| 11 | RN | 1 | 901.8 | 76.0 | 11,733.8 |
| 12 | RN | 1 | 619.1 | 76.0 | 8,342.2 |
| 13 | RN | 1 | 501.3 | 63.4 | 6,775.7 |
| 14 | EN | 1 | 494.9 | 50.7 | 6,547.5 |
| 15 | EN | 1 | 346.6 | 50.7 | 4,768.1 |
| 16 | EN | 1 | 354.2 | 44.4 | 4,783.3 |
| 17 | EN | 1 | 494.9 | 50.7 | 6,547.5 |
| 18 | EN | 1 | 501.3 | 31.7 | 6,395.4 |
| 19 | EN | 1 | 346.6 | 63.4 | 4,920.2 |
| 20 | Attendant | 1 | 216.7 | 31.7 | 2,981.0 |
| 21 | Attendant | 1 | 139.7 | 38.0 | 2,133.1 |
| 22 | Manager | 1 | 914.4 | 158.4 | 12,874.5 |
|  |  |  |  |  |  |
| 2. Out-Patient Department | | |  |  |  |
| 1 | MO | 1 | 708.2 | 95.1 | 9,639.5 |
| 2 | MO | 1 | 708.2 | 95.1 | 9,639.5 |
| 3 | AMO | 1 | 747.8 | 95.1 | 10,114.1 |
| 4 | CO | 1 | 539.3 | 95.1 | 7,612.2 |
| 5 | AMO | 1 | 737.6 | 95.1 | 9,992.4 |
| 6 | AMO | 1 | 728.0 | 95.1 | 9,876.8 |
| 7 | Intern | 1 | 551.3 | 76.0 | 7,528.5 |
| 8 | AMO | 1 | 757.7 | 95.1 | 10,232.7 |
| 9 | AMO | 1 | 698.4 | 95.1 | 9,520.9 |
| 10 | Intern | 1 | 485.4 | 76.0 | 6,737.6 |
| 11 | Intern | 1 | 485.4 | 76.0 | 6,737.6 |
| 12 | CO | 1 | 507.6 | 95.1 | 7,231.9 |
| 13 | AMO | 1 | 619.1 | 95.1 | 8,570.3 |
| 14 | EN | 1 | 342.8 | 63.4 | 4,874.5 |
| 15 | EN | 1 | 358.0 | 63.4 | 5,057.0 |
| 16 | EN | 1 | 346.6 | 63.4 | 4,920.2 |
| 17 | EN | 1 | 343.5 | 63.4 | 4,882.1 |
| 18 | EN | 1 | 343.5 | 63.4 | 4,882.1 |
| 19 | EN | 1 | 339.0 | 63.4 | 4,828.9 |
| 20 | EN | 1 | 354.2 | 63.4 | 5,011.4 |
| 21 | EN | 1 | 507.6 | 50.7 | 6,699.6 |
| 22 | Attendant | 1 | 301.0 | 31.7 | 3,992.4 |
| 23 | Attendant | 1 | 228.1 | 31.7 | 3,117.9 |
| 24 | Manager | 1 | 889.1 | 158.4 | 12,570.3 |
|  |  |  |  |  |  |
| 3. Pharmacy Department | | |  |  |  |
| 1 | Pharmacist | 1 | 952.5 | 50.7 | 12,038.0 |
| 2 | Pharmacist | 1 | 952.5 | 38.0 | 11,885.9 |
| 3 | Pharmacist | 1 | 589.7 | 44.4 | 7,609.1 |
| 4 | Pharmacy Technician | 1 | 611.8 | 25.3 | 7,645.6 |
| 5 | Pharmacy Technician | 1 | 342.8 | 15.8 | 4,304.2 |
| 6 | Pharmacy Technician | 1 | 350.4 | 22.2 | 4,471.5 |
| 7 | Pharmacy Assistant | 1 | 346.6 | 9.5 | 4,273.8 |
| 8 | Intern | 1 | 399.2 | 25.3 | 5,095.1 |
| 9 | Attendant | 1 | 220.5 | 9.5 | 2,760.5 |
| 10 | Attendant | 1 | 158.1 | 9.5 | 2,011.4 |
|  |  |  |  |  |  |
| 4. Laboratory Department | | |  |  |  |
| 1 | Principle Lab technician | 1 | 939.8 | 76.0 | 12190.1 |
| 2 | Lab technician | 1 | 361.9 | 22.2 | 4608.4 |
| 3 | Lab technician | 1 | 365.7 | 22.2 | 4654.0 |
| 4 | Lab Technician | 1 | 231.9 | 22.2 | 3049.4 |
| 5 | Lab technician | 1 | 354.2 | 22.2 | 4517.1 |
| 6 | Lab technician | 1 | 342.8 | 22.2 | 4380.2 |
|  | | | | | |

Key:

MD-Medical Officer ( 5 years degree training)

AMO-Assistant Medical Office (3 years diploma training+2 years of upgrading)

CO-Clinical Officer (3 year diploma training)

RN-Registered Nurse (3 years degree or diploma training)

EN-Enrolled Nurse (2 year certificate training

Attendant-nurse with one year certificate training

B: Overhead personnel costs

| 1. Administration department | | | | |
| --- | --- | --- | --- | --- |
| No | Cadre of personnel | Monthly Gross salary | Monthly allowances | Annual earnings |
| 1 | Medical officer | 1,136.88 | 253.49 | 16,684.41 |
| 2 | Office secretary | 228.01 | 12.67 | 2,888.21 |
| 3 | Office secretary | 228.01 | 19.01 | 2,964.26 |
| 4 | IT specialist | 249.81 | 0.00 | 2,997.72 |
| 5 | IT specialist | 249.81 | 0.00 | 2,997.72 |
| 6 | Chief accountant | 324.08 | 95.06 | 5,029.66 |
| 7 | Assistant accountant | 222.56 | 63.37 | 3,431.18 |
| 8 | Assistant accountant | 222.56 | 63.37 | 3,431.18 |
| 9 | Office supervisor | 450.38 | 63.37 | 6,165.02 |
| 10 | Assistant office supervisor | 450.38 | 63.37 | 6,165.02 |
| 11 | Hospital Secretary | 1,207.22 | 158.43 | 16,387.83 |
| 12 | Clinical Service coordinator | 1,207.22 | 158.43 | 16,387.83 |
| 13 | Supportive manager | 977.82 | 158.43 | 13,634.98 |
| 14 | Records coordinator | 140.68 | 63.37 | 2,448.67 |
| 15 | Office attendant | 153.68 | 50.70 | 2,452.47 |
| 16 | Office attendant | 153.68 | 50.70 | 2,452.47 |
| 17 | Office attendant | 153.68 | 50.70 | 2,452.47 |
| 18 | Matron | 708.24 | 158.43 | 10,400.00 |
| 19 | Health officer | 927.12 | 126.74 | 12,646.39 |
| 20 | Procurement officer | 168.06 | 95.06 | 3,157.41 |
| 21 | Procurement officer | 370.22 | 95.06 | 5,583.27 |
| 28 | Salaries casual labourers | Contracted |  | 13,464.83 |
| 30 | Civil works | Contracted |  | 70,082.54 |

| 2. Transportation Department | | | | |
| --- | --- | --- | --- | --- |
| No |  | Monthly salary Gross | Monthly allowances | Annual earnings |
| 1 | Driver 1 | 198.35 | 63.37 | 3,140.68 |
| 2 | Driver 2 | 167.30 | 63.37 | 2,768.06 |
| 3 | Driver 3 | 189.48 | 63.37 | 3,034.22 |
| 4 | Driver 4 | 198.35 | 63.37 | 3,140.68 |
| 5 | Driver 5 | 176.17 | 63.37 | 2,874.52 |
| 6 | Driver 6 | 176.17 | 63.37 | 2,874.52 |

C: Building rental costs

| **No.** | **Building** | **Area (m^2^)** | **Rental charges per year** |
| --- | --- | --- | --- |
| 1 | General Outpatient | 100.00 | 9,125.48 |
| 2 | Paediatric ward | 218 | 19,893.54 |
| 3 | Laboratory | 127.3 | 11,616.73 |
| 4 | Pharmacy | 190.25 | 17,361.22 |
| 5 | Administration | 476 | 43,437.26 |
